# Chest radiographs of cardiac devices (Part 1): Cardiovascular implantable electronic devices, cardiac valve prostheses and Amplatzer occluder devices

**DOI:** 10.4102/sajr.v23i1.1730

**Published:** 2019-07-31

**Authors:** Rishi P. Mathew, Timothy Alexander, Vimal Patel, Gavin Low

**Affiliations:** 1 Department of Radiology and Diagnostic Imaging, Faculty of Medicine and Dentistry, University of Alberta, Edmonton, Canada

**Keywords:** Chest radiographs, pacemaker, implanted cardioverter defibrillators, cardiac resynchronisation therapy, implantable loop recorder, valve replacement, transcatheter valve replacement, amplatzer septal occluder, amplatzer ductal occluder

## Abstract

Several new innovative cardiac devices have been created over the last few decades. Chest radiographs (CXRs) are the most common imaging investigations undertaken because of their value in evaluating the cardiorespiratory system. It is important for the interpreting radiologist to not only identify these iatrogenic objects but also to assess for their accurate placement, as well as for any complications related to their placement, which may be seen either on the immediate post-procedural CXR or on a follow-up CXR.

## Introduction

Over the last few decades, several new innovative cardiac medical devices have been created. Almost all of the patients with implanted cardiac devices such as pacemakers, implantable cardioverter defibrillators (ICDs), cardiac resynchronisation therapy (CRT) devices, implantable loop recorders (ILR) and cardiac prosthetic valves undergo chest radiographs (CXRs) on a regular basis. Therefore, it is not uncommon for the resident, radiologist, intensivist or physician to be presented with a conundrum of CXRs having a variety of these devices on a day-to-day basis. Chest radiographs are the initial modality for evaluating the device location and its integrity after implantation and for diagnosis of complications and malfunction.^1^ The intention of this article is to inform the readers about these cardiac devices, their indications, their proper position on CXRs and commonly associated complications.

## Cardiovascular implantable electronic devices

Cardiovascular implantable electronic devices (CIEDs) include implantable cardiac pacemakers, ICDs, CRT devices (also known as biventricular devices) and implantable cardiac monitors. In 2012 alone, at least 3 million patients were implanted with CIEDs.^2^ In the United States, approximately 100 000 ICDs and 300 000 pacemakers are implanted annually,^3^ while in the UK, the 10-year average growth rate for pacemakers and ICDs is 4.7% and 15%, respectively.^4^ It is important to understand the basic functions and differences between various CIEDs, as well as to recognise them on a radiograph.

**FIGURE 1:**
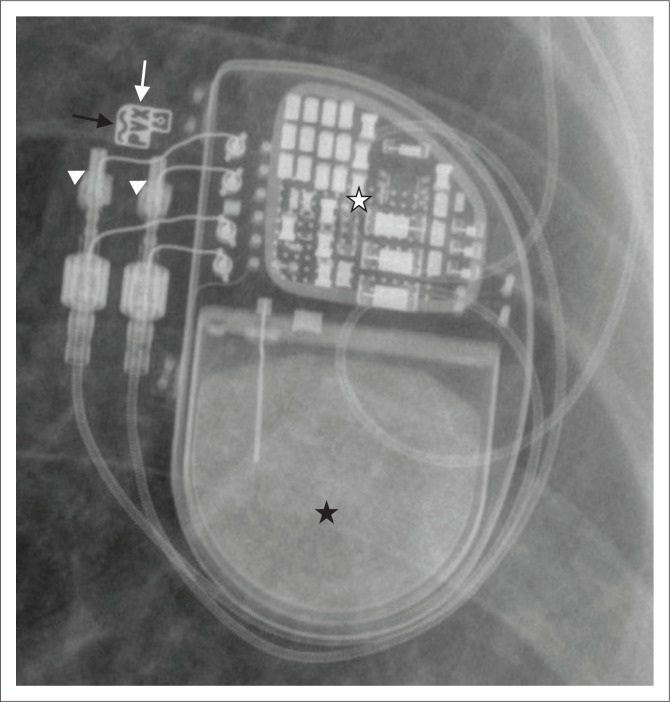
Basic components of a cardiovascular implantable electronic device (CIED) generator include the battery (black star), the circuitry (white star) and the terminal connector ports (arrow heads). The pacemaker generator contains the manufacturer logo (white arrow). This pacemaker is MRI conditional as indicated by the curvilinear line (black arrow) near the manufacturer logo.

The CIED is composed of two main components: the pulse generator encased in titanium and the pacemaker or ICD lead(s). The pulse generator (Figure 1) comprises the circuitry, a lithium battery and the connector port. The CIED lead has five major parts: a conductor, insulation (silicone rubber or polyurethane), electrode(s), a fixing mechanism and a terminal connector pin. Proximally the leads are connected to the generator by the terminal connector by means of a connector block.^5^ Lead tips can be fixed actively or passively. Leads placed passively have radiolucent ‘tines’ at their end that anchor the lead tips in position. With time, the myocardium surrounding the lead tip undergoes fibrosis, further securing the lead tip in place. Active fixation leads have a retractable screw at its end which is deployed when the lead is placed in position. Specific situations where active fixation is used include right ventricular (RV) leads in the outflow tract and in situations where the right atrial (RA) leads must be secured to the tissue for stability. Active fixation leads are more commonly used in younger patients as it can be extracted much easier than passive fixation leads.^6^

### Pacemakers

A pacemaker is a medical device that regulates the heart rate by electrical impulses delivered through electrodes to the heart muscles. The primary aim of this device is to maintain an adequate heart rate so as to not fall below a certain limit (mostly 60 beats/min), either because of a dysfunction of the heart’s natural pacemaker or because of a block in the electrical conduction system of the heart.^4,5^ Pacemakers can be temporary or permanent.

#### Temporary pacemakers

These pacemakers are meant for short-term use during hospitalisation such as for bradydysrhythmia following a heart attack, cardiac surgery or drug overdose. The main equipment of the temporary pacemaker (the external pacemaker generator) is located outside the body and may be attached to the patient’s skin by a tape or body by a belt.^6^ The method of choice for temporary pacing in the intensive care unit (ICU) is transvenous pacing. Temporary epicardial pacing is the most common choice following cardiac surgery and for permanent pacing in children. Other alternatives include transthoracic, transesophageal or transcutaneous pacing routes.^7,8^

#### Permanent transvenous pacemakers

**FIGURE 2:**
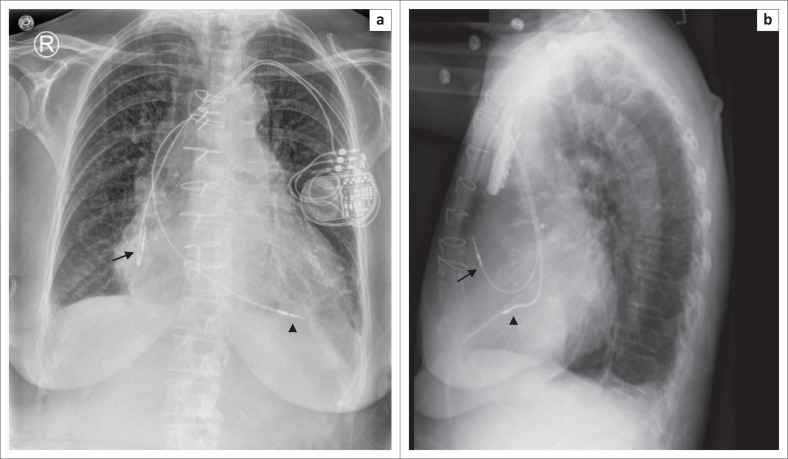
Frontal (a) and lateral (b) view chest radiographs showing a dual-chamber pacemaker with the right atrial lead (arrow) in the right atrial appendage forming a ‘J’ on the lateral view and the right ventricular lead (arrow head) pointing towards the cardiac apex.

**FIGURE 3:**
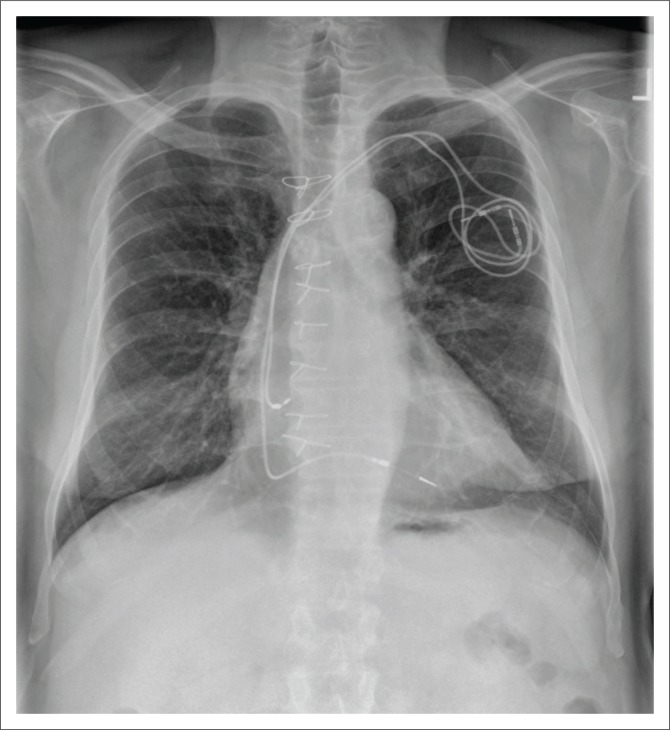
A chest radiograph (CXR) showing abandoned pacemaker leads in the right atrium and the right ventricle.

**FIGURE 4:**
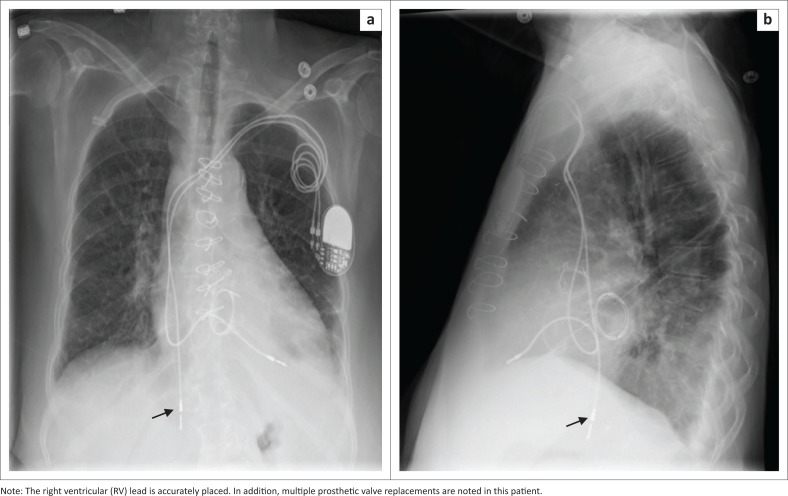
Frontal (a) and lateral (b) view chest radiographs (CXRs) showing a dual-lead pacemaker with the right atrial lead (arrow) abnormally positioned in the inferior vena cava.

These pacemakers are meant for chronic cardiac rhythm dysfunction with abnormally low heart rate and, in general, have a sensing (and/or pacing) lead in the right atrium and a pacing (and/or sensing) lead in the right ventricle. Permanent pacemakers can be single-chamber pacemakers (usually with a single lead in the apex of the right ventricle especially for patients with atrial fibrillation; however, a single lead may be seen in the atrium for patients with sick sinus syndrome), dual-chamber pacemakers (with two leads, one in the RA appendage and the second in the RV apex) (Figure 2a and b) or biventricular pacemakers also known as CRT devices that have been developed for treating severe congestive heart failure. The CRT device will be discussed later separately.^1,8,9^ The leads of the permanent pacemakers are inserted using a transvenous route usually through the left or right subclavian vein or rarely through the internal jugular, axillary or femoral vein. The pulse generator is implanted into the subcutaneous layer of chest below the clavicle and above the pectoral muscle (prepectoral fascia). Rarely, the pulse generator may be placed under the skin of the abdomen, inframammary site in women for cosmetic reasons and right infraclavicular site for the left handed.^4,10^ During the insertion of a pacemaker, the standard procedure is a fluoroscopic evaluation of the positioning of the pacemaker electrodes, followed up by a CXR post insertion. Chest radiographs are also useful for identifying abandoned pacemaker leads (Figure 3) and complications (Figure 4a and b) which have been elaborated on in detail in Table 1.^4,10^

**TABLE 1:** How to assess cardiac implantable electronic devices on chest radiographs.

Step	How to assess
Step1: Look for immediate post-procedural complications:	Look for immediate post-procedural complications, for example, myocardial perforation (ventricular lead located in abnormal location such as subdiaphragmatic location, pericardial/pleural effusion, cardiac tamponade or extracardiac stimulation of the diaphragm, intercostal or abdominal muscles), pneumothorax and haemothorax.
Step2: Differentiate between a pacemaker and an ICD.	An ICD comprises of a single lead with one or two shock coils. As these shock coils are radiopaque, they can be readily identified on a CXR, enabling ICDs to be differentiated from a pacemaker.
Step 3: Evaluate the electrode port insertion sites connecting to the generator.	The proximal end of an electrode has an insertion port that attaches the lead to the generator. The electrode should slightly extend beyond the connector for the device to function properly. If not, repositioning of the electrode will be required.
Step 4: Look for lead damage or breakage by tracing their entire course.	Normally, the lead should follow a linear pathway without forming any loops, as it may cause cardiac arrhythmia and even migration and translocation of the lead tip. In patients with normal anatomic variants such as left SVC or congenital cardiac conditions (e.g. patent foramen ovale and transposition of the great arteries), the leads may show an abnormal course or location of their ends. It is not uncommon to see CXRs with abandoned leads because fibrous tissue around the abandoned leads can make their removal unsafe (Figure 3). The incidence of lead breakages is < 5% and can occur anywhere along its pathway. The commonest sites are at their attachment with the generator or at the entry of the subclavian vein, where it gets crushed between the clavicle and first rib (also known as clavicle crush or subclavian crush). Electrode fracture occurs when there is a break in continuity between two extremes (Figure 9).
Step 5: Confirm that the electrode tip is accurately positioned and does not change on follow-up CXRs.	The RA lead should be in the RA appendage. On a posterior–anterior (PA) radiograph, the RA lead has a slight medial course, while on a lateral CXR, its location is anterior subtending an angle <90 and forming a ‘J’. The RV lead on a PA CXR has its tip pointing towards the cardiac apex and should be to the left of the spine, while on the lateral view, the lead should curve along the course of the right atrial lateral wall, passing the tricuspid valve and reaching the apex of the heart, pointing anteriorly and slightly superiorly (or inferiorly). Alternative placement sites in the RV include the outflow tract. In patients with normal anatomic variants such as left SVC or congenital cardiac conditions (e.g. patent foramen ovale and transposition of the great arteries), the leads may show an abnormal course or location of their ends.^5,10^
	In patients with CRT devices, it is normal to see an electrode in the coronary sinus (left ventricular [LV] electrode), and for it to be accurately placed in the coronary sinus, the electrode tip needs to be at the posterolateral coronary vein, anterior interventricular vein or the middle cardiac vein. On a frontal radiograph, it is difficult to differentiate an RV electrode from an LV one. For this purpose, a lateral CXR can be more useful. In this case, the RV electrode is located anteriorly in the lateral segment, while the LV electrode is located in the posterior segment of the cardiac silhouette. Although the above-mentioned positions are the optimal location for the electrodes, some amount of variation is permissible as long as the obtained potentials are good. Hence, it is always advisable to compare a follow-up CXR with prior ones to identify any change in position of the electrode(s) indicating dislocation or migration.^10^
Step 6: Identify if the CIED is MRI conditional.	By evaluating the pacemaker generator, the radiologist can determine if the implanted pacemaker is magnetic resonance imaging (MRI) conditional thereby contributing to patient management by determining whether an MRI is the best imaging study to answer a clinical question (if it is indicated), as well as suggesting ‘absolute’ contraindications for non-MRI conditional CIEDs which include CIED implanted <6 weeks prior, CIED with retained or fractured device leads, surgically placed permanent epicardial pacing leads and temporary intracardial or epicardial pacer devices with the external generator still attached. Intubated or heavily sedated patients are considered as a relative contraindication. However, these contraindications are slightly disputed by some experts.
Step 7: Evaluate the correct position of the pacemaker casing inside the pocket and look for complications.	Complications that can occur at the pacemaker pocket are hematoma, infections and presence of air-fluid level. Two syndromes are associated with abnormal rotation of the pacemaker generator, namely Twiddler’s syndrome and Reel syndrome. In Twiddler’s syndrome, the casing is rotated along the axis of its leads, usually as a result of the patient’s own manipulation, causing the leads to get twisted around the casing, leading to displacement at their ends. Reel syndrome is a variant of the Twiddler’s syndrome, where the casing rotates along its sagittal axis.

ICD, implantable cardioverter defibrillators; SVC, superior vena cava; RA, right atrial; RV, right ventricular; LV, left ventricular; CIED, cardiac implantable electronic devices; CXR, chest radiographs; CRT, cardiac resynchronisation therapy; MRI, magnetic resonance imaging.

Recently, leadless pacemaker systems have been developed as a minimally invasive option for patients requiring single-chamber pacemaker placement. The two RV leadless pacemaker systems currently available for clinical use are the Nanostim™ Leadless Pacemaker System (LPS) (St. Jude Medical) and the Micra™ Transcatheter Pacing System (TPS) (Medtronic), both of which are inserted via femoral venous access and implanted directly into the RV wall. On CXRs, these devices appear as a linear radiopaque material implanted into the RV wall. Potential complications that can be identified on CXRs include device dislodgment and cardiac perforation.^11^

### Implanted cardioverter defibrillators

**FIGURE 5:**
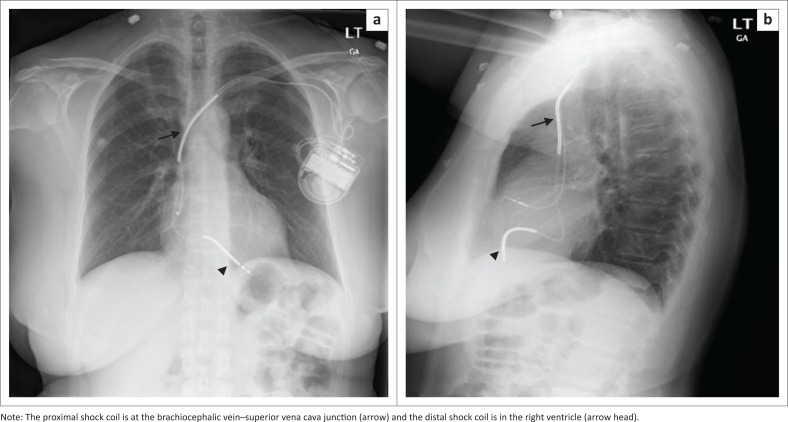
Chest radiographs posterior–anterior (a) and lateral views (b) showing a dual-lead implantable cardioverter defibrillator with its characteristic shock coils implanted in a 62-year-old female patient with non-ischaemic cardiomyopathy.

**FIGURE 6:**
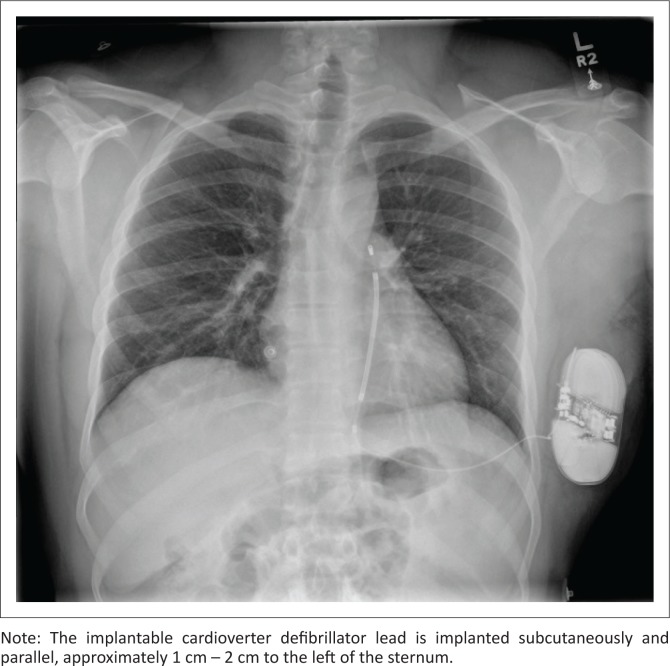
A subcutaneous implantable cardioverter defibrillator implanted in a 27-year-old male patient with hypertrophic cardiomyopathy.

An ICD is a medical device capable of producing a large amount of electrical energy in a single output, used to defibrillate the heart. It is mainly used in patients with tachydysrhythmias (e.g. ventricular tachycardia or ventricular fibrillation) and for preventing cardiac arrest. An ICD usually comprises of a single lead with one or two shock coils. A shock coil has a relatively thick electrode to reduce the risk of damage to the myocardium from the defibrillating shock. When a two-shock-coil device is placed accurately, one shock coil will be terminating at the brachiocephalic vein–SVC junction and the second coil in the RV (Figure 5a and b). As these shock coils are radiopaque, they can be readily identified on a CXR, enabling ICDs to be differentiated from a pacemaker.^5,10,12^ A newer approach is to place a subcutaneous ICD lead to the left of the sternum (Figure 6).

**FIGURE 7:**
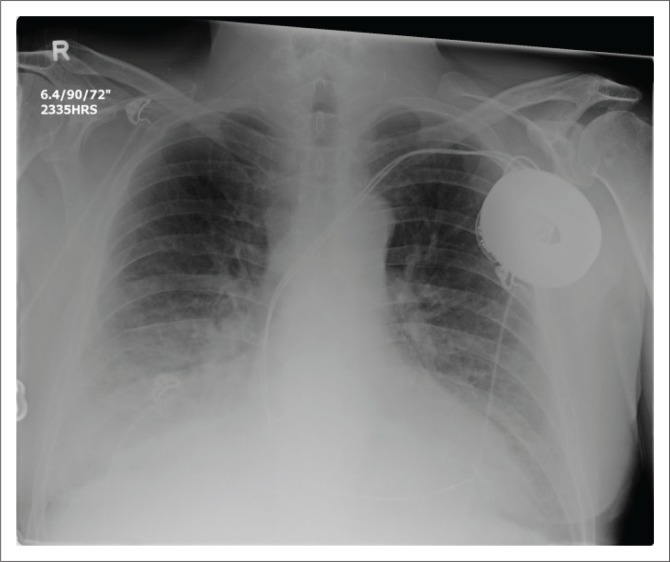
A chest radiograph showing a doughnut magnet placed over a pacemaker.

During surgery in the operating room, a CIED can sense an external magnetic interference (EMI) that can affect its pacing function. The source of the EMI may include electrocautery, external defibrillation, radio frequency ablation (RFA) and dental instruments. A cardiac device magnet (Figure 7) may be used temporarily by placing it securely and directly over the CIED generator to switch the CIED to asynchronous pacing mode and hence prevent the EMI from interfering with the device pacing. Once the magnet has been removed, the CIED will revert to its original programmed settings.^9^

**FIGURE 8:**
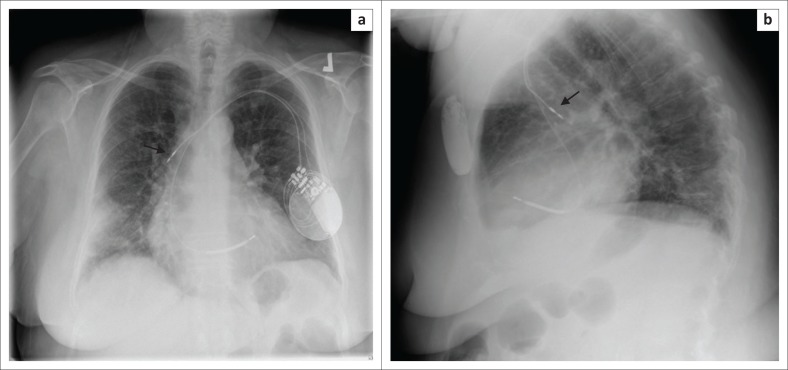
Posterior–anterior (a) and lateral (b) chest radiograph views showing a dual-lead implantable cardioverter defibrillator with a single right ventricular shock coil. Note its right atrial lead (arrow) malpositioned in the superior vena cava.

**FIGURE 9:**
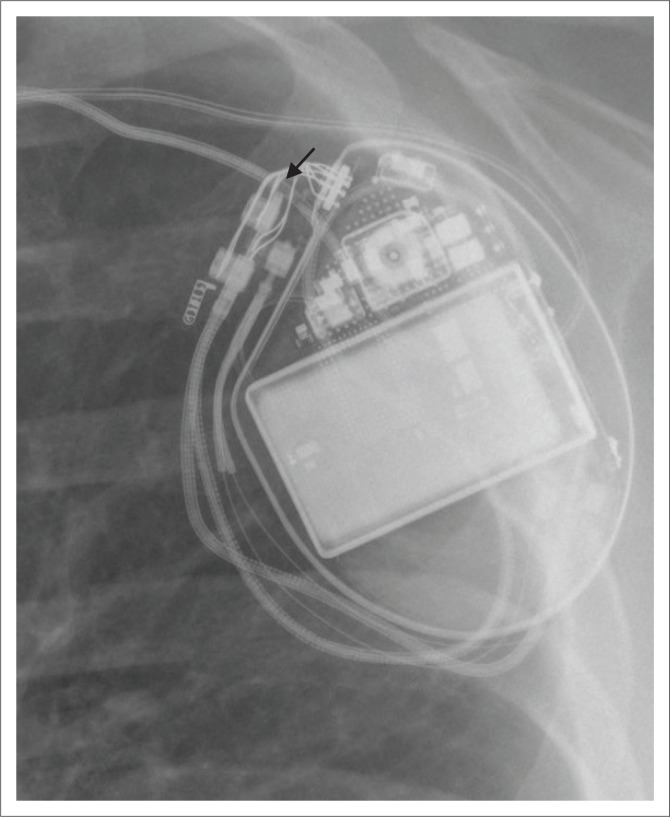
A dual-lead implantable cardioverter defibrillator with a subtle fracture (arrow) in the proximal portion of the right atrial lead, close to the generator.

**FIGURE 10:**
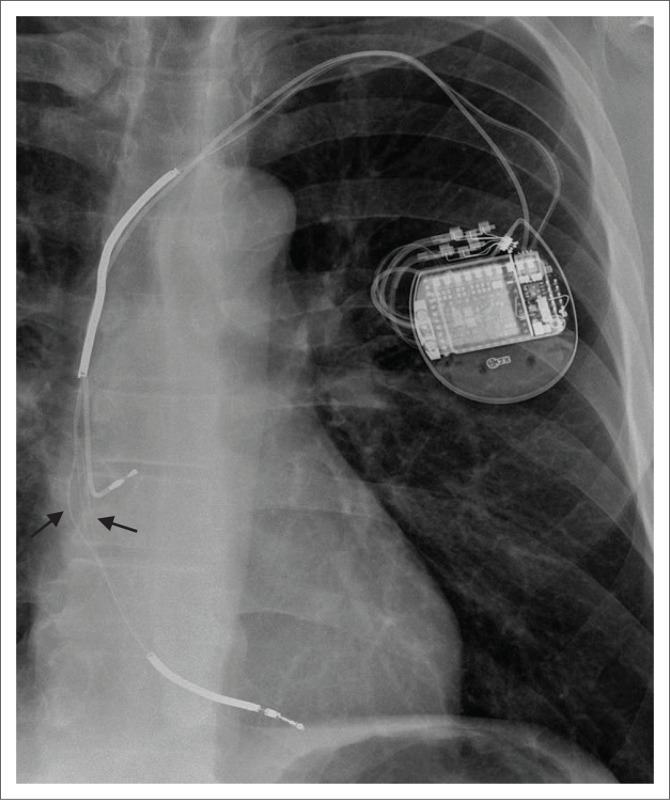
A dual-lead implantable cardioverter defibrillator showing externalised conductors (arrows) of its right ventricular lead, leading to its failure.

A detailed stepwise approach to the assessment of an ICD including its associated complications (Figures 8a and b, 9 and 10) on a CXR is like a pacemaker and is elaborated on in Table 1.

### Cardiac resynchronisation therapy devices (implantable cardioverter defibrillator and pacemaker combinations)

**FIGURE 11:**
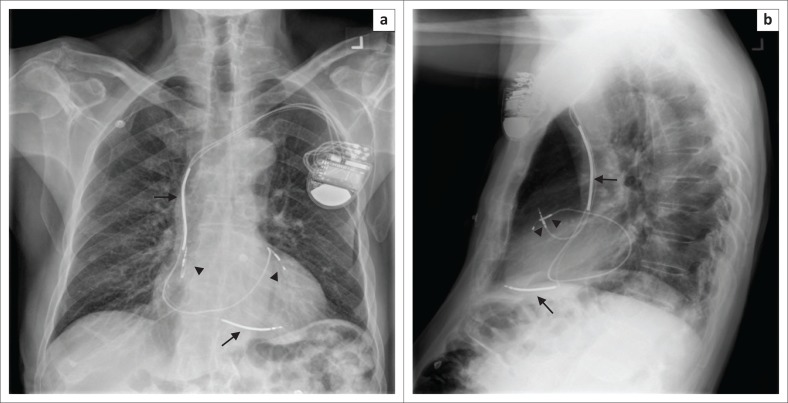
Frontal (a) and lateral (b) chest radiographs showing a cardiac resynchronisation therapy device with its pacemaker leads (arrow heads) in the right atrial appendage and the left ventricle, and its implantable cardioverter defibrillator shock coils (arrows) in the superior vena cava and right ventricular apex.

ICD and pacemaker leads can be used in various combinations to produce a CRT device. The most common combination is a biventricular pacemaker with an ICD (Figure 11a and b), where the two pacemaker leads are noted in the right atrium and LV, while the shock lead of the ICD is in the RV. Cardiac resynchronisation therapy devices are mainly used for treating congestive heart failure (CHF). The CRT device can be more commonly an ICD (CRT-D) or a standalone pacemaker (CRT-P). The main aim of this device, in addition to a being pacemaker or ICD, is to synchronise the left ventricular contraction to improve the symptoms of CHF.^5,10,12^ The assessment of a CRT device on a CXR is no different from the other CIEDs.

By closely evaluating the CIED generator, a radiologist can determine if the device is MR conditional, as there are several ‘absolute’ contraindications to performing magnetic resonance imaging (MRI) in patients implanted with non-MR conditional CIEDs (elaborately detailed in Table 1),^13^ although some of these contraindications remain slightly disputed.^14^

### Insertable cardiac monitor or implantable loop recorder

**FIGURE 12:**
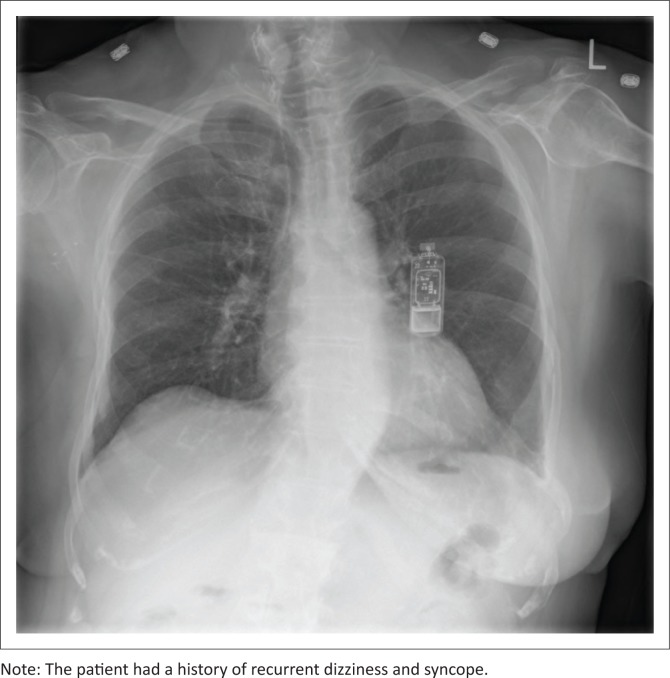
Chest radiograph of a 67-year-old female patient with an implantable loop recorder.

An insertable cardiac monitor (ICM) or ILR (Figure 12) is a miniaturised subcutaneous electrocardiographic monitoring device that has been extensively used for evaluating patients with unexplained syncope, symptomatic palpitations in adults, transient or occult atrial fibrillation (AF) and in the evaluation of a stroke.^12^ When compared to pacemakers and ICDs, ICMs do not require central venous access or direct contact with the endocardium, thereby negating the risk for endocardial infection. The device is generally implanted in the subcutaneous tissue overlying the left pectoralis muscle with a 1 cm – 2 cm incision that does not require conscious sedation. On a CXR, the ICM appears like a USB drive with no wires or leads attached to it. Insertable cardiac monitor mimics on a PA CXR include a USB flash drive, an e-vape device and a leadless cardiac pacemaker in the RV, which can be differentiated from them on a lateral CXR.^1,15^

## Cardiac valves and valve replacements or repair

**FIGURE 13:**
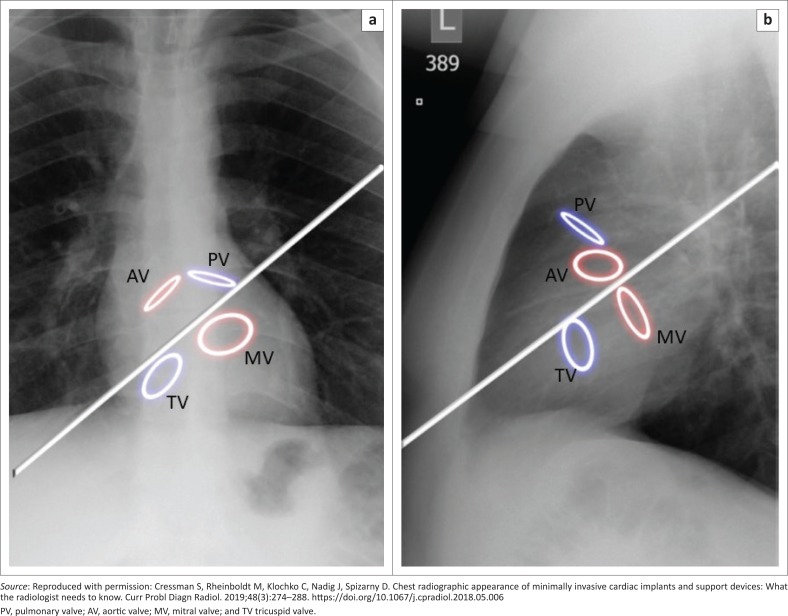
By drawing an imaginary line extending from the left atrial appendage to the right cardiophrenic angle on a posterior–anterior (a) chest radiograph and an imaginary line from the carina to the cardiac apex on the lateral view (b), the cardiac valves can be identified.^16^

Landmarks of the cardiac silhouette and of the thorax can enable the identification of heart valves on a CXR. By drawing an imaginary line extending from the left atrial appendage to the right cardiophrenic angle (Figure 13)^16^ on a PA CXR and a line extending from the carina to the apex on the lateral view, positions of the valves can be roughly estimated. The pulmonic valve is the most superiorly positioned valve in both views, appearing in partial profile on both projections. On both views, the aortic valve is located superior to the axis line, appearing in profile and in front on the frontal and lateral views, respectively. In contrast, the mitral valve is located inferior to the axis lines on both views, appearing en face on the frontal projection and in profile on the lateral view. The tricuspid valve is located the most inferiorly on both projections, appearing anteriorly and en face on the lateral view. Nevertheless, because of anatomical and physiological variations and differences in radiographic projections, valve identification based solely on CXR can be unreliable, necessitating the need for additional details such as clinical history.^16^

**FIGURE 14:**
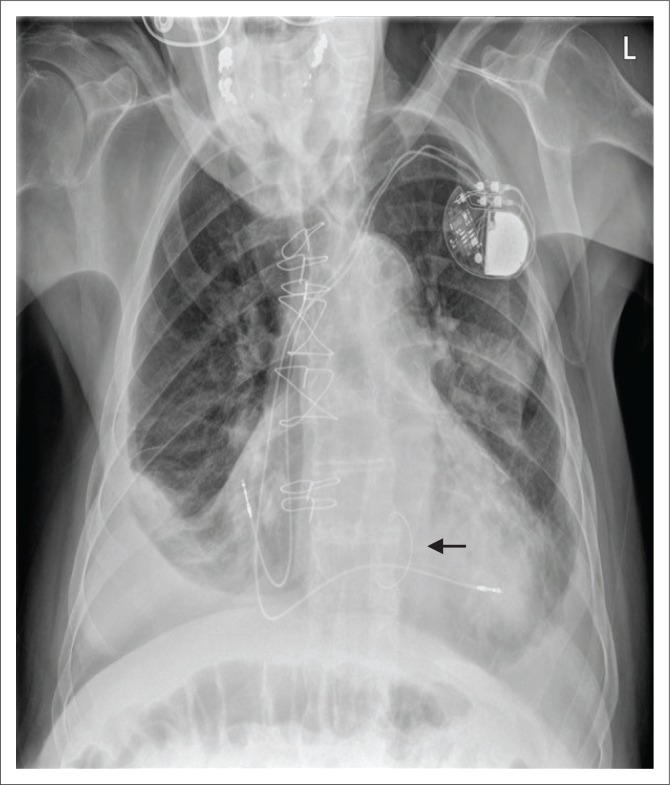
Chest radiograph showing a mitral annuloplasty C ring (arrow). Note the dual-lead pacemaker with accurately placed right atrial and right ventricular leads.

**FIGURE 15:**
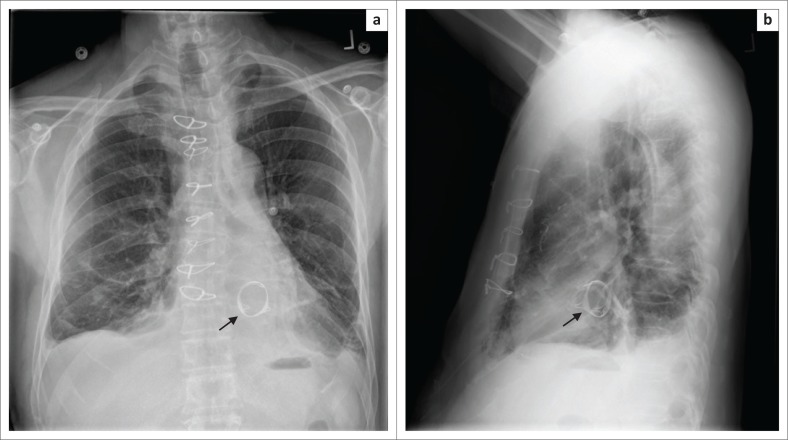
Frontal (a) and lateral (b) chest radiographs showing mitral (perimount tissue) valve replacement (arrow) in a patient with severe mitral stenosis.

**FIGURE 16:**
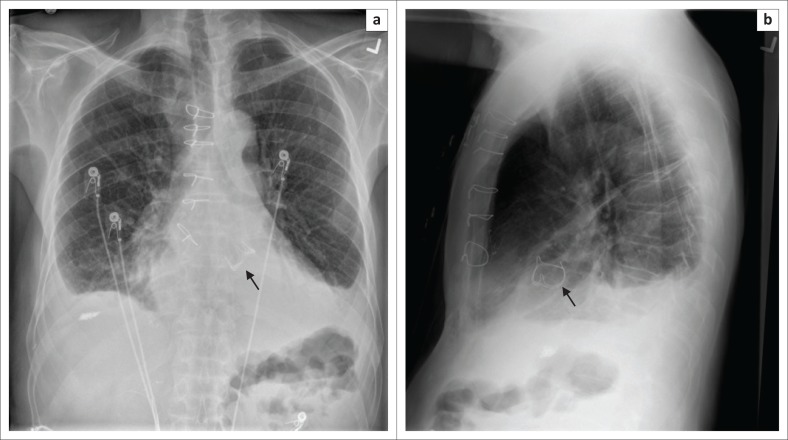
Frontal (a) and lateral (b) chest radiographs showing an aortic valve (perimount magna ease tissue valve) replacement (arrow) in a 76-year-old male patient with severe aortic stenosis.

**FIGURE 17:**
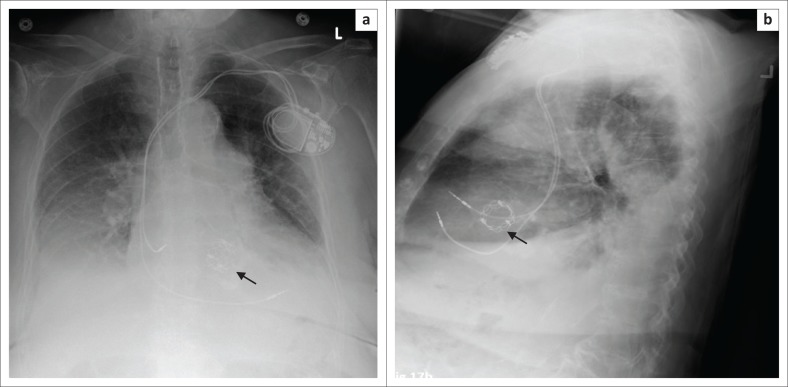
Frontal (a) and lateral (b) chest radiographs showing transcatheter aortic valve replacement (arrow) in an 81-year-old female patient with severe aortic stenosis.

**FIGURE 18:**
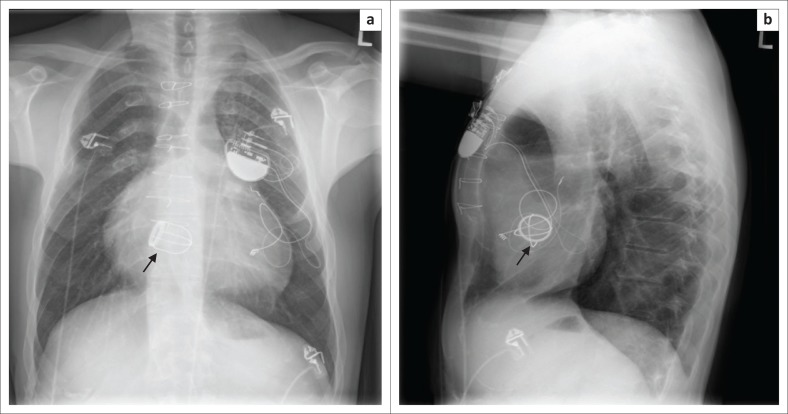
Frontal (a) and lateral (b) chest radiographs showing an isolated prosthetic tricuspid valve replacement (Starr Edwards valve) (arrow) in a 51-year-old male patient with a history of Ebstein’s anomaly. Note the implanted pacemaker with its epicardial leads.

**FIGURE 19:**
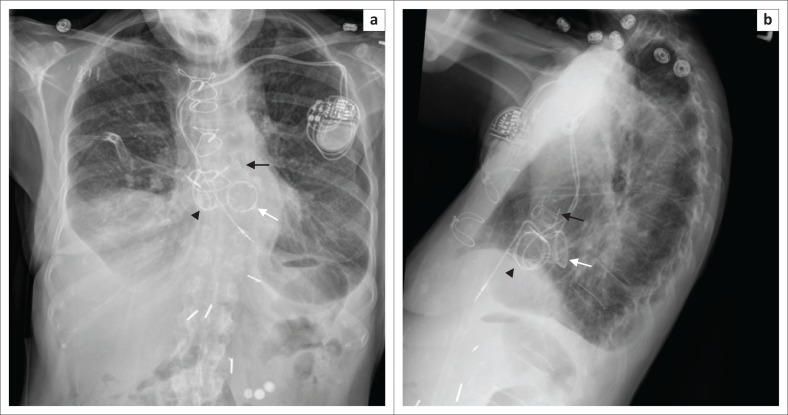
Frontal (a) and lateral (b) chest radiographs showing prosthetic aortic (black arrow), mitral (white arrow) and tricuspid (arrow head) valves in a 72-year-old female patient with a history of aortic and mitral stenosis, tricuspid regurgitation and complete heart block.

**FIGURE 20:**
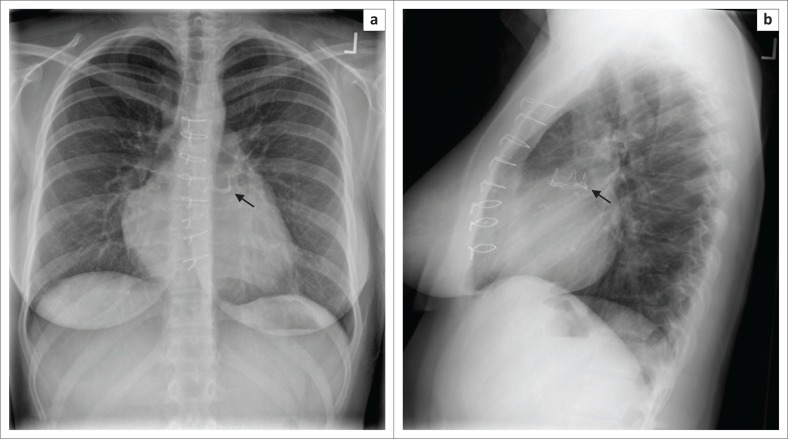
Frontal (a) and lateral (b) chest radiographs (CXRs) showing prosthetic pulmonary valve replacement (arrows).

Valvular heart disease is associated with significant morbidity and mortality and affects roughly more than 100 million people worldwide, and the current standard of management is surgical valve replacement (Figures 14–20). Alternatively, transcatheter valve replacement without surgery has been gaining popularity over the last decade, especially in patients not suitable for surgery. Based on the leaflet material, the two types of prosthetic heart valves available are the mechanical and biological or bioprosthetic heart valves (BHVs).^17^ The various types of biological and mechanical prosthetic valves have been highlighted in Table 2, and they all look slightly different on imaging. The choice between mechanical valve and bioprosthetic valve remains controversial and is based on multiple parameters (Tables 2 and 3).^18,19^

**TABLE 2:** Types of prosthetic heart valves.

Heart valve	Type	Prosthetic
Biological or bioprosthetic heart valves (BHVs)	Stented	Porcine bioprosthesis
		Pericardial bioprosthesis
	Stentless	Porcine bioprosthesis
		Pericardial bioprosthesis
		Aortic homograft
		Pulmonary autograft (Ross procedure)
	Sutureless	-
	Transcatheter	-
Mechanical heart valves	Bileaflet	-
	Single tilting disk	-
	Caged ball	-

**TABLE 3:** Bioprosthetic and mechanical heart valves: advantages and disadvantages.

Prosthetic valve	Advantages	Disadvantages
Bioprosthetic	No need for lifelong anticoagulation Lower risk for bleeding Can be used in patients contraindicated for anticoagulation, for example, pregnancy Preferred in older patients, patients with cancer and renal failure on haemodialysis	Shorter durability and hence higher re-operation rates
Mechanical	Longer durability and lesser re-operation rates Desirable haemodynamic properties: low gradients and low disturbances in flow Preferred in younger patients Preferred in patients with already another mechanical heart valve	Increased risk of thrombosis Lifelong need for anticoagulation Increased risk of bleeding

Identification of the prosthetic heart valve on a CXR is made possible by detecting any or all its radiopaque parts which may include the base ring, stent, struts, cage, ball or disc. In some cases, a prosthesis may have no radiopaque component and hence cannot be identified radiographically. In most cases, the base ring is radiopaque. Several prosthetic valves have radiopaque struts or stents above or below the base ring. If the projections are long, the prosthetic heart valve is either a ball-in-cage or bioprosthesis. Literature sources are available,^20^ providing detailed algorithms guiding in the identification of prosthetic heart valves on CXRs which is beyond the scope of this article.

A stepwise approach to the evaluation of CXRs following cardiac valve replacement is elaborated in Table 4.^21^

**TABLE 4:** A stepwise approach for the evaluation of chest radiographs following cardiac valve replacement.

Step	Evaluation of the cardiac valve(s)
Step 1	Identification of the valve(s) replaced.
Step 2	Identify the number of valve(s) replaced.
Step 3	Evaluate the position and orientation of the valve(s) replaced.
Step 4	As valve replacement is most often done through a median sternotomy, look for changes in the cardiac and mediastinal contour.
Step 5	Evaluate for post-valve replacement complications: Once the native valves have been replaced, a radiologist must bear in mind complications such as paravalvular insufficiency (structural changes of the base ring, struts or occlude), features of obstruction (thrombus formation or tissue overgrowth), thromboembolism, infective endocarditis and sequelae of chronic anticoagulant use, for example, pulmonary and gastrointestinal haemorrhage. On follow-up CXRs, look for the presence or absence of calcifications of the valve, coronary artery or cardiac wall, evidence of pulmonary artery or vein hypertension and pleural pathology.
Step 6	Evaluate the ribs for rib fractures or rib separations from right or left thoracotomy (e.g. a prior mitral commissurotomy may show a separation or fractured left posterior 5th and 6th ribs, a right thoracotomy may be because of a prior lung surgery or mitral or tricuspid plication, etc.).

CXRs, chest radiographs.

## Amplatzer septal occluder and Amplatzer ductal occluder

**FIGURE 21:**
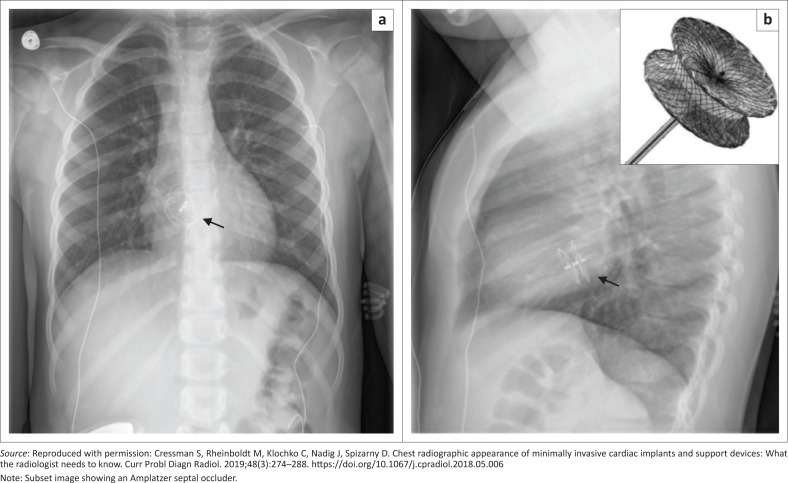
Chest radiographs showing a normally positioned Amplatzer septal occluder (arrow) in a 4-year-old girl with a history of a secundum atrial septal defect. Note that the ASO is projecting over the right lateral margins of the T8 vertebral body on the frontal view (a) and located anterior to the hilar-caval line on the lateral view (b).^16^

The Amplatzer septal occluder (ASO) (AVP, AGA Medical Corp., Golden Valley, MN, USA) is the most commonly used device for transcatheter closure of secundum atrial septal defects. The location of the ASO on a CXR is based on the position of the secundum atrial septal defect (ASD). On frontal CXRs (Figure 21a), the ASO is projected to the right or over the spinous processes between the T7 and T9 vertebral bodies, and on lateral CXRs (Figure 21b), the ASO is projected anterior to or over the hilar-caval line.^22^ ASO migration or embolisation is a recognised complication with an incidence of 0.4% – 1.1%, which can be identified on a CXR. Common sites of migration are the right cardiac chambers and pulmonary artery, while left-sided migration is rare.^23^

**FIGURE 22:**
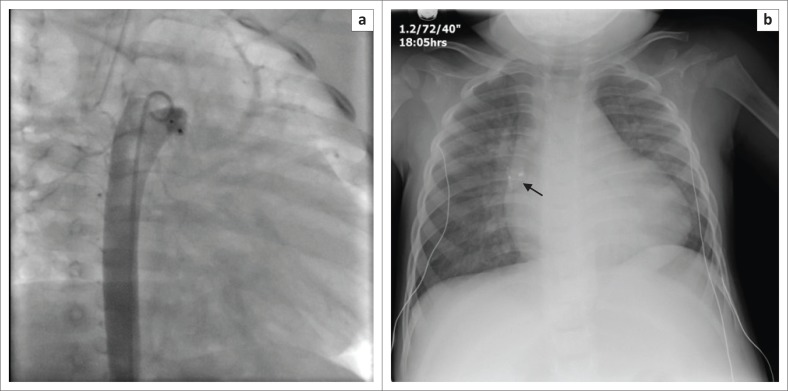
A migrated amplatzer duct occluder placed for patent ductus arteriosus. Aortogram in the oblique anterior–posterior view (a) shows transcatheter deployment of the amplatzer duct occluder in the lumen of the aorta. Post-procedural chest radiograph (b) shows migration of the device (arrow).

The Amplatzer ductal occluder (ADO) is a mushroom-shaped self-expandable nitinol wire mesh used for occluding large patent ductus arteriosus (PDA), while smaller PDAs are occluded by the transcatheter method using Gianturco coils. A recognised complication with the ADO, like the ASO, is device embolisation or migration (Figure 22a and b).^24^

## Ethical consideration

All ethical considerations have been taken into account. No patient identity or patient information has been revealed.

## Conclusion

As more and more patients are living with CIEDs and prosthetic valves, it is important that residents, radiologists and physicians are aware of these devices and recognise them on a CXR. Radiologists in particular have a specific and important role in the evaluation of these devices. An early and accurate identification of device malfunction, fracture or migration can help prompt the physician or surgeon to intervene in a timely manner and help avoid preventable life-threatening catastrophes.
