# Erratum: Chest radiographs of cardiac devices (Part 1): Cardiovascular implantable electronic devices, cardiac valve prostheses and Amplatzer occluder devices

**DOI:** 10.4102/sajr.v25i1.2256

**Published:** 2021-11-17

**Authors:** Rishi P. Mathew, Timothy Alexander, Vimal Patel, Gavin Low

**Affiliations:** 1 Department of Radiology and Diagnostic Imaging, Faculty of Medicine and Dentistry, University of Alberta, Edmonton, Canada

In the version of this article initially published, Mathew RP, Alexander T, Patel V, Low G. Chest radiographs of cardiac devices (Part 1): Cardiovascular implantable electronic devices, cardiac valve prostheses and Amplatzer occluder devices. S Afr J Rad. 2019;23(1), a1730. https://doi.org/10.4102/sajr.v23i1.1730, the article section was given incorrectly. The correct section should be Review Article instead of Original Research.

This correction does not alter the study’s findings of significance or overall interpretation of the study’s results. The publisher apologises for any inconvenience caused.

